# Oral Kaposi sarcoma development is associated with HIV viral load, CD4+ count and CD4+/CD8+ ratio

**DOI:** 10.4317/medoral.24708

**Published:** 2021-10-27

**Authors:** Rosa Hiolanda Abreu de Sousa, Lucas Lacerda de Souza, Pablyanne Tereza Louzada Guedes, Ana Carolina Prado-Ribeiro, Leticia Rodrigues-Oliveira, Thaís Bianca Brandão, Barbara Waleria Gonçalves Alves, Márcio Ajudarte Lopes, Alan Roger Santos-Silva, Julius Caesar Mendes Soares Monteiro, Thaís Tapajós Gonçalves, Oslei Paes de Almeida, Flavia Sirotheau Correa Pontes, Hélder Antônio Rebelo Pontes

**Affiliations:** 1 Oral Diagnosis Department (Pathology and Semiology), Piracicaba Dental School University of Campinas (UNICAMP), Brazil; 2 Oral Pathology Department, João de Barros Barreto University Hospital (JBBUH), Federal University of Pará (UFPA), Belém, Brazil; 3 Dental Oncology Service, Instituto do Câncer do Estado de São Paulo (ICESP-FMUSP), Brazil; 4 Infectious and Parasitic Diseases Department, João de Barros Barreto University Hospital (JBBUH), Federal University of Pará (UFPA), Belém, Brazil; 5 General Surgery Department, João de Barros Barreto University Hospital (JBBUH), Federal University of Pará (UFPA), Belém, Brazil

## Abstract

**Background:**

Kaposi’s sarcoma (KS) is an uncommon, multifocal and angioproliferative lesion, which demonstrates a poor prognosis. The aim of the present research was to explore the association of HIV viral load, CD4+ and CD8+ counts and the CD4+/CD8+ ratio on the risk of oral Kaposi’s sarcoma (KS) development.

**Material and Methods:**

A total of 62 patients were retrieved from March 2008 to October 2020 from the files of two oral pathology centres. Clinical, laboratory and follow-up data were retrieved from their medical files. Poisson regression was used to explore the role of history of immunosuppression and its association with oral KS development. A *P-value* <0.05 was considered significant.

**Results:**

Sixty-two patients were included in the present study (32 with oral KS and 30 with no presentation of lesions anywhere on the body). Patients with oral KS presented a mean age of 32.6 years, and male patients were more affected. The hard palate (15 cases; 46.8%) was the main anatomical site affected. The lesions were mostly presented as swellings (13 cases; 40.6%) and nodules (12 cases; 37.5%). Systemic manifestations were also observed, including candidiasis (4 cases; 12.5%), bacterial infection (3 cases; 9.3%), tuberculosis (3 cases; 9.3%), herpes simplex (3 cases; 9.3%) and pneumonia (3 cases; 9.3%). A significant correlation was observed between HIV viral load, CD4+ count and the CD4+/CD8+ ratio with oral KS development.

**Conclusions:**

HIV viral load, CD4+ count and the CD4+/CD8+ ratio are associated with oral KS development.

** Key words:**Cancer, oral, Kaposi’s sarcoma, diagnostic.

## Introduction

Kaposi’s sarcoma (KS) is an uncommon, multifocal, angioproliferative lesion initially described by Moritz Kaposi in 1872 (1,2). The tumour is formed by the endothelial cells of blood and lymphatic vessels and shows a variety of clinical, epidemiological and immunophenotypic characteristics (3,4). KS was classified epidemiologically by Antman and Chang in 2000: 1) the classic form occurs in middle-aged or elderly patients, 2) the endemic type is presented in Sub-Saharan Africa, 3) the epidemic category is AIDS associated and 4) the iatrogenic type is related to immunosuppression in patients receiving anti-rejection therapy for transplanted organs (5,6). The aetiology of KS is associated with human herpesvirus-8 (HHV8) in all epidemiologic subtypes of the lesion (7,8).

KS is recognized as an AIDS-defining cancer, along with non-Hodgkin’s lymphoma and invasive cervical cancer (3,8,9). Clinically, lesions may be presented in the skin, oral mucosa, gastrointestinal tract, lymph nodes and lungs (8,10). When the oral cavity is affected, lesions can demonstrate a variable morphology, from plaques to swellings with a purple or dark-brown appearance (6,11,12). In addition, KS was identified as the second most common sarcoma of the oral cavity according to a multicentre study of oral sarcomas in the Brazilian population (11).

The prognosis for people living with HIV/AIDS (PLWHA) improved with the initiation of highly active antiretroviral therapy (HAART) in the mid ‘90s (13-15). Despite significant advances in KS therapy, the innate immune system do not contributes significantly to treatment effectiveness (3-5). HIV viral load (HVL), CD4+ (CD4L) and CD8+ (CD8L) levels and the CD4+/CD8+ (CD4L/CD8L) ratio are all very important in the assessment of the patients’ systemic condition (8,10,16,17).

According to previous literature, the association of patients’ systemic condition and the development of oral KS has not been well established. Thus, the objective of this study is to evaluate the association of HVL, CD4L and CD8L and the CD4L/CD8L ratio with the development of oral KS.

## Material and Methods

- Study design and ethical approval

This research was developed on the files from two oral pathology centres of Brazil. Samples were retrieved from the centres over a period of 12 years (from March 2008 to October 2020). The diagnosis centres were João de Barros Barreto University Hospital, Federal University of Pará, Belém/Brazil and Instituto do Câncer do Estado de São Paulo (ICESP-FMUSP), São Paulo/Brazil. Expert oral pathologists from each centre evaluated the samples. The ethics committee of the João de Barros Barreto University Hospital approved this work (No. 3.952.288). The patients’ identities remained anonymous according to the Declaration of Helsinki.

- Samples

Patients from both control and KS groups were retrieved based on the initial diagnosis of HIV/AIDS. KS in the oral cavity were recovered, and data regarding sex, age, location, clinical aspects, HIV/KS diagnosis and laboratory findings (HVL, CD4L, CD8L and CD4L/CD8L) were retrieved. Ranges of laboratory findings were classified following Taiwo & Hassan (2010), following: HIV viral load was ranged as <20, 20≤199, 200≤999 and >999 copies/mL; CD4+ as ≤200, 201-499 and ≥500 cells/mm3; CD8+ as ≤150, 150-1000 and >1000 cells/mm3; CD4+/CD8+ ratio was considered according to the mean values of all patients (18). In cases that oral KS was not confirmed, when patients refused to participate the research and when laboratory tests were not assessed represented the exclusion criteria. Lesions were diagnosed following the methods of our study group (19). For better illustration, all cases were also stained with CD34, D2-40 (podoplanin) and Prox-1.

- Data analysis

Means and percentages are presented as descriptive statistics. Poisson regression was used to explore the role of the prevalence of immunosuppression and the association of oral KS development, exploring HVL, CD4L, CD8L and CD4L/CD8L ratio. A P-value <0.05 was considered statistically significant. Data were analysed using IBM SPSS Statistics for Windows, version 23.0 (Armonk, NY).

## Results

A total of 62 HIV-positive patients, including 32 patients with clinical presentation of oral KS and 30 patients with no KS lesions on the body were included in this research.

**Figure 1:**
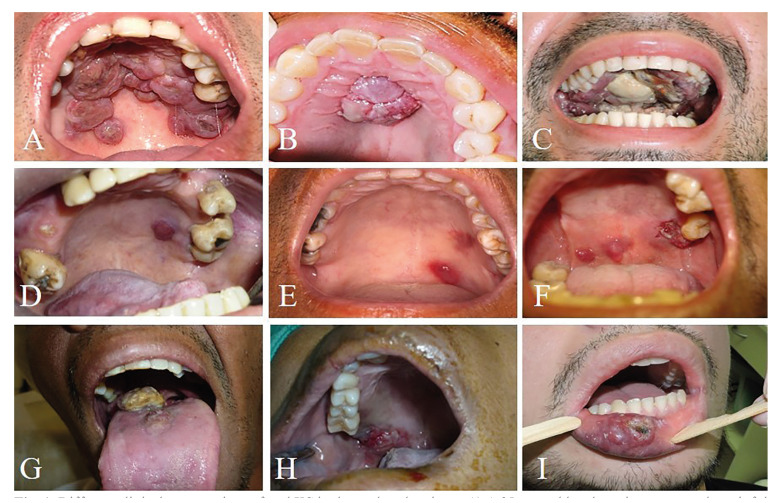
Different clinical presentations of oral KS in the analysed patients. A) A 35-year-old male patient presented a painful swelling in the hard palate with superficial areas of necrosis. B) A 39-year-old male patient with a nodular lesion in the hard palate. C) A 31-year-old male patients demonstrated extensive bleeding and a necrotic lesion in the hard palate. D) A 58-year-old male patient presented a bleeding nodule in the hard palate. E) A 30-year-old male patient showed an asymptomatic purple spot in the hard palate. F) A 25-year-old male patient was presented with a necrotic lesion and bleeding in the hard palate associated with posterior teeth, as well as two nodules in the soft palate. G) A 23-year-old male patient demonstrated a bleeding and painful swelling in the posterior tongue with necrotic areas. H) A 41-year-old male patient was referred with an ulcerated swelling in the hard palate. I) A 25-year-old male patient presented with a painful and ulcerated lesion in the lower lip.

Regarding patients with oral KS, a mean age of 32.6 years (range of 19–58 years old) was observed. Male patients were mostly affected, with a M:F ratio of 10.6:1. The hard palate (15 cases; 46.8%) was the main location of the lesion, followed by the alveolar ridge (6 cases; 18.7%), soft palate (6 cases; 18.7%), tongue (5 cases; 15.6%) and gums (5 cases; 15.6%). The mandible (2 cases; 6.2%), maxilla (1 case; 3.1%), lower lip (1 case; 3.1%) and upper lip (1 case; 3.1%) were less frequently affected. Lesions were presented as swellings (13 cases; 40.6%), nodules (12 cases; 37.5%), plaques (5 cases; 15.6%) and spots (2 cases; 6.2%). They showed purple (53.1%) and red colouration (15 cases; 46.8%) (Fig. 1). In addition, oral KS showed bleeding in 15 cases (46.8%) and pain in 14 cases (43.7%). The lesions were the first manifestation of HIV/AIDS in 23 cases (71.8%). Patients also showed lesions in the skin (10 cases; 31.2%), intestine (4 cases; 12.5%), stomach (4 cases; 12.5%), trachea (3 cases; 9.3%), lung (3 cases; 9.3%), eyes (1 case; 3.1%) and pharynx (1 case; 3.1%). Systemic comorbidities were observed in 16 cases (50%) and oral candidiasis (4 cases; 12.5%), bacterial infection (3 cases; 9.3%), tuberculosis (3 cases; 9.3%), oral herpes simplex (3 cases; 9.3%), pneumonia (3 cases; 9.3%), gastroenteritis (2 cases; 6.2%), syphilis (2 cases; 6.2%) and meningitis (2 cases; 6.2%) were most often seen.

**Figure 2:**
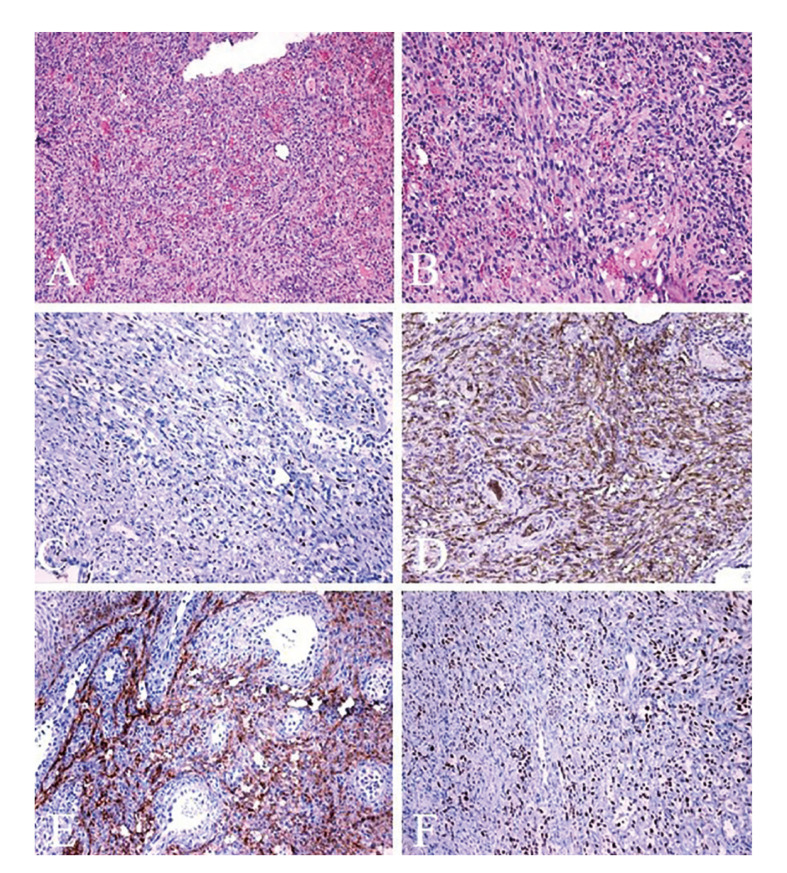
Histopathological and immunohistochemistry analysis of oral KS. A) Proliferation of spindle cells intermixed with numerous congested blood vessels (H&E, 100x). B) Spindle cells with significant pleomorphism and presence of blood-filled slits like spaces (H&E, 200x). Immunohistochemistry reaction showing positivity for HHV-8 (DAB, 200x) (C), CD34 (DAB, 200x) (D), D2-40 (podoplanin) (DAB, 200x) (E) and Prox-1 (DAB, 200x) (F).

Histopathological findings showed fascicular arrangement of spindle cells and extravasation of red blood cells. It was also observed blood filled slits like spaces, spindle shaped cells with prominent pleomorphism. Immunohistochemistry reactions were positive for CD34, D2-40, Prox-1 and HHV-8 in situ hybridization (Fig. 2).

The control group had a mean age of 41.6 years (range of 25–61 years), and male patients were more frequently affected than females, with a M:F ratio of 1.3:1. Oral manifestation was observed in 5 patients (16.6%), and candidiasis (3 cases; 10%), oral herpes (2 cases; 6.6%) and hairy leukoplakia (1 case; 3.3%) were the manifestations seen. Systemic comorbidities were observed in all cases, and tuberculosis (14 cases; 46.6%), meningitis (5 cases; 16.6%), neurotoxoplasmosis (5 cases; 16.6%), bacterial infection (4 cases; 13.3%), scabies (2 cases; 6.6%), pneumopathy (2 cases; 6.6%) and syphilis (2 cases; 6.6%) were the main presentations.

Laboratory findings of patients who presented oral KS evidenced a mean HVL of 149,487.6 copies/mL (range of 0–1,556,502 copies/mL), mean CD4L count of 155.9 cells/mm3 (range of 2–837 cells/mm3), mean CD8L count of 944.8 cells/mm3 (range of 143–3056 cells/mm3) and mean CD4L/CD8L of 0.17 cells/mm3 (range of 0.01–0.70 cells/mm3). The control group showed a mean HVL of 592,295.6 copies/mL (range of 69–6,254,071 copies/mL), mean CD4L count of 112.5 cells/mm3 (range of 10–540 cells/mm3), mean CD8L count of 996.6 cells/mm3 (range of 229–4557 cells/mm3) and mean CD4L/CD8L count of 0.16 cells/mm3 (range of 0.01–0.57 cells/mm3) (Table 1).

Statistical analysis evidenced by the Poisson regression test for prevalence analysis that HVL (HR 95% CI: 1.6517 [1.4681–4.2033]; *p*<0.0001), CD4L (HR 95% CI: 2.8058 [0.0667–8.5103]; *p*<0.0001) and CD4L/CD8L ratio (HR 95% CI: 1.7613 [0.7740–2.1304]; *p*<0.0001) were significantly associated with oral KS development (Table 2).

**Table 1:**
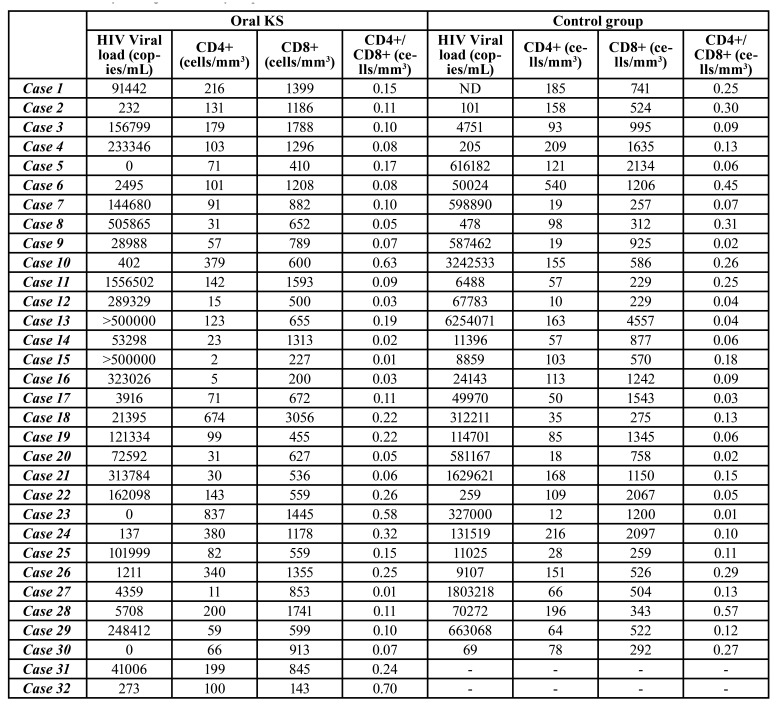
Laboratory findings of the analysed patients.

**Table 2:**
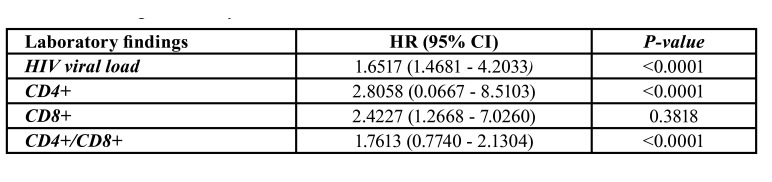
Poisson regression analysis.

## Discussion

Clinical and pathological information regarding oral KS is still very limited due to the diverse clinical presentation and because lesions are mostly presented in late manifestation of HIV/AIDS (4,11,19). The complete aetiopathogeneses of oral KS remains unclear, although HHV-8 represents the main etiological agent (20). The patient’s immune system is evaluated by laboratory analysis of HVL, CD4L, CD8L and the CD4L/CD8L ratio, and their influence on clinical disease manifestation has been widely discussed (3,21). Thus, the objective of the study was to correlate HVL, CD4L, CD8L and the CD4L/CD8L ratio and their influence on oral KS development.

Clinically, oral KS demonstrated a male predominance, similar to previous literature (5,16). The lesions were most commonly presented in young patients and rarely affect elderly individuals, despite the increase in HIV/AIDS infection and the incidence of oral KS in patients aged 50 and over (22). When presented in the oral cavity, KS is most frequently diagnosed in the hard and soft palate (2,11). Oral tumours may present a wide diversity of presentations, varying from single spots to bleeding, painful and necrotic swellings (2,19). They also may present colour alteration in the buccal mucosa due to their angiogenic origin, ranging from red to purple lesions (5,7,8,19).

The systemic affliction of HIV/AIDS patients generally causes, besides the oral cavity, development of KS in the skin, gastrointestinal and respiratory complex, consistent with the findings of the present study (22). Systemic conditions commonly associated with immunocompromised patients were also observed, including oral presentation of oral candidiasis and herpes simplex, as well as systemic diseases including tuberculosis, pneumonia, gastroenteritis, syphilis and meningitis (6,18,19).

Medical follow-ups of PLWHA generally involve a multidisciplinary assessment based on laboratory findings, including HVL, CD4L, CD8L and CD4L/CD8L, and other complementary exams when necessary (23,24). However, an increase in the number of reports of oral KS as the first manifestation of HIV/AIDS has been shown, consistent with the findings of our study that 71.8% of patients showed oral lesions as the first manifestation of the disease (16,17).

Thus, laboratory exams may be presented as a good option to evaluate patients’ systemic condition (8,10,15). The goal of this study was to explore the association of HVL, CD4L, CD8L and CD4L/CD8L ratio and oral KS development compared with a control group with no lesions on the body. Interestingly, HVL, CD4L and CD4L/CD8L ratio showed significant results when compared with the control group, demonstrated significant weight of the association of these variables with KS development. Rezende *et al*. (25) observed a relationship of HVL with AIDS-related KS, resulting in a significant relationship of HVL and upper gastrointestinal KS, consistent with our findings. Many studies have explored the association of CD4L cell count with AIDS-related KS, and they showed a significant correlation of KS development when a CD4L ≤200 cells/mm3 was observed, similar to our results (26,27). In addition, Poizot-Martin *et al*. (26) reported that a CD4L/CD8L ≤0.5 increased the risk of development of KS, corroborating our results as we showed a significant higher probability of developing oral KS with altered CD4L/CD8L levels. Hence, the clinical significance of HVL, CD4L count and the CD4L/CD8L ratio is noteworthy in the development of oral KS.

More recently, KS genomics has gained attention over the past decades due to its remarkable pathogenic mechanisms. The association of the HHV-8 genome and KS development has been explored worldwide (27). It has been shown that more than 80 genes are expressed in the regulated transcriptional program that promotes latency with very limited viral expression or supports lytic replication with the production of progeny virions (28,29). The cellular tropism of HHV-8 in KS includes epithelial, endothelial and B cells and more recently has been expanded to include neurons (29,30).

The present study showed a significant result of oral KS development and its association with HVL, CD4L count and the CD4L/CD8L ratio. Despite the limitations on the number of patients to validate the current results, this is the first study to explore laboratory findings and oral KS development as an alternative method to improve diagnostic accuracy. Additionally, knowledge of the influence of other etiologic factors is important to better establish the etiopathogenesis and pathogenesis of the disease, as well as to determine the gene alterations related to KS development.
